# Supplementary material for: Cerebellar activity in PINK1 knockout rats during volitional gait
Source: Brain Commun. 2024 Oct 25;6(5):fcae249. doi: 10.1093/braincomms/fcae249 (PMC11503944; doi:10.1093/braincomms/fcae249)
Supplement: fcae249_Supplementary_Data [file fcae249_supplementary_data.pdf]

## Supplementary Material

**Supplementary Table 1: Interlobular coherence.** Coherence was lowest in the delta band when compared to other frequency bands in WT rats. P-values of these interactions range from <0.001 to 0.03.

| Lobular Pair | Pairwise Comparisons (a>b) | | p-value |
|---|---|---|---|
| | a | b | |
| **VIa-VIb** | theta | delta | 0.030 |
| | high beta | | 0.022 |
| | low gamma | | 0.005 |
| | high gamma | | <0.001 |
| | fast freq. | | <0.001 |
| **VIa-VIc** | alpha | delta | 0.005 |
| | low beta | | 0.003 |
| | high beta | | <0.001 |
| | low gamma | | <0.001 |
| | high gamma | | <0.001 |
| | fast freq. | | <0.001 |
| **VIa-VII** | high beta | delta | 0.029 |
| | low gamma | | 0.020 |
| | high gamma | | 0.003 |
| | fast freq. | | <0.001 |
